# Selected Immunohistochemical Assessment and Clinical Examinations in the Diagnosis of Palatine Tonsil Diseases

**DOI:** 10.3390/jcm12134522

**Published:** 2023-07-06

**Authors:** Przemysław Bant, Dariusz Jurkiewicz, Szczepan Cierniak

**Affiliations:** 1 Department of Otolaryngology and Cranio-Maxillo-Facial Surgery, Military Institute of Medicine—National Research Institute, 04-141 Warsaw, Poland; djurkiewicz@wim.mil.pl; 2 Department of Pathomorphology, Military Institute of Medicine—National Research Institute, 04-141 Warsaw, Poland; scierniak@wim.mil.pl

**Keywords:** palatine tonsillitis, tonsillar hypertrophy, immunohistochemical assessment, diagnosis, clinical assessment, pathomorphological assessment

## Abstract

Introduction: The palatine tonsils are secondary lymphoid organs where immune processes occur, influencing the development of a targeted cellular and humoral response. The diseased tonsils are subject to immunological imbalances, including the activity of pro-inflammatory and anti-inflammatory factors. This leads to the development of palatine tonsil diseases, such as palatine tonsillitis and palatine tonsillar hypertrophy. Aim: The main aim of the study was to evaluate the similarities and differences in the clinical and pathomorphological pictures of patients qualified for surgical treatment due to hypertrophy or inflammation of the palatine tonsils. The aim was achieved by demonstrating the relationship between the patient’s medical history and physical examination and histopathological diagnosis of a given tonsillar disease, evaluating the usefulness of basic blood tests (leukocytosis, ASO, ESR, and CRP) in differential diagnosis, and assessing the immunohistochemical assessment of palatine tonsil tissue. Material and Methods: The tonsils were stained with the following antibodies: IL-1, IL-2, IL-6, IL-8 IL-10, and IL-37 and CD25, CD40, and CD69, taking into account the histological division of the studied lymphatic tissue (epithelial, subepithelial, follicular, follicular center, and interfollicular). Patients aged between 19 and 70 years with tonsillitis or clinical signs of tonsillar hypertrophy were qualified for tonsillectomy/UPPP. Seventy-two males (68.6%) and thirty-three females (31.4%) were enrolled in the study. Histopathological and immunohistochemical assessment was performed on 105 palatine tonsils. Results: The diagnostic value of blood tests, including determination of ASO, ESR, CRP, and leukocyte level, proved to be a significant predictor of tonsil disease. In the pathomorphological assessment, 75% of the subjects who had simultaneously elevated ESR (>4.73) and leukocytosis (>6.96) and reduced ASO (<161.03) and CRP (<0.31) belonged to the tonsillitis group. The immunohistochemical assessment revealed a diverse profile of the markers tested depending on the diagnosed disease of the tonsils. The follicular center proved to be the region of palatine tonsil tissue for which the most statistically significant differences between the markers were found. Responses to CD-40 and IL-1 were observed in this region. The tissue of epithelial, follicular, and interfollicular regions each showed one statistically significant value for the studied chemokines and lymphokines. However, the lack of significant statistical differences for *p* < 0.05 between the study groups was only noted in the subepithelial region. It should be emphasized that for the data as a whole (calculated on the basis of the data for all regions together), no statistically significant differences were observed. Conclusion: In conclusion, the results obtained are indicative of the presence of a specific immunohistochemical profile for palatine tonsil diseases. Significant discrepancies have been found in the clinical and pathomorphological assessment of tonsils qualified for tonsillectomy. Therefore, these methods should be considered complementary. The patient’s medical history and physical examination, depending on the adopted clinical or histopathological classification, show a variation in the distribution of features that are the basis for allocation to a particular group.

## 1. Introduction

The palatine tonsils are secondary lymphoid organs containing aggregates of lymphoid cells, and they belong to the mucosa-associated lymphoid tissue (MALT). The pharyngeal mucosa has a complex secretory immune system. B cells are initially stimulated in MALT regions, and the resulting activated lymphocytes migrate to glandular sites, where they differentiate into Ig-producing cells [1]. Therefore, the proliferation, differentiation, and stimulation of B lymphocytes are the main functions of the palatine tonsils and are essential in the processing of the specific immune response [2]. It should be emphasized that B lymphocytes represent more than 50% of the tonsillar lymphocyte population compared to their lower percentage found in blood and other lymphoid organs [3,4]. Leukocytes, mainly lymphocytes, are present in all regions of the tonsils, including the lymphatic epithelium, where they are called intraepithelial leukocytes (IEL). The number of IELs in patients with tonsillitis is significantly higher than in patients with tonsillar hypertrophy. This is due to a selective increase in the CD8 T-cell population Vdelta1/Vgamma9 [5]. CD4+ T lymphocytes constitute a smaller proportion of the tonsillar IELs than the CD8+ population, the former occurring with B lymphocytes in the epithelium of the crypts [6]. The B lymphocytes of the tonsils are mainly found in three locations: in the follicular mantle, in the germinal centers, and as intraepithelial cells [7]. The epithelium lining the crypts contains dendritic cells that can transport exogenous antigens to extracellular areas of T cells and to B-cell follicles [8]. The resulting interleukins are a range of secretory proteins that function as cytokines, harmonizing intercellular communication during a complex immune response [9]. In conclusion, the induction of the immune response involves specific immunological reactions in the tonsils in response to several different processes in which cytokines play an integral role [10,11,12]. There may be an increased activity of pro-inflammatory as well as anti-inflammatory factors in the affected palatine tonsils. In our study, we sought to verify the research hypothesis of whether pro-inflammatory factors predominate in palatine tonsillectomy and anti-inflammatory factors in palatine tonsillar hypertrophy. For the study, we selected those cytokines that appear to be most relevant in the pathogenesis of palatine tonsil disease in the literature. Therefore, we analyzed the following: CD25, CD40, CD69, IL-1, IL-2, IL-6, IL- 8, IL-10, and IL37 [13,14,15,16,17,18].

## 2. Materials and Methods

Patients aged between 19 and 70 years with inflammation of the palatine tonsils or clinical signs of palatine tonsillar hypertrophy were qualified for tonsillectomy. Seventy-two men (68.6%) and thirty-three women (31.4%) participated in the study. The mean age of the men and women was 42 and 35 years, respectively. Histopathological and immunohistochemical assessment was performed on 105 palatine tonsils resected due to tonsillitis or tonsillar hypertrophy at the Department of Otolaryngology and Laryngological Oncology with the Clinical Department of Cranio-Maxillofacial Surgery of the Military Institute of Medicine—National Research Institute in Warsaw. Tissues showing a typical microscopic structure, undamaged during the surgical procedure, were selected as diagnostic from a pathomorphological point of view.

Each patient’s medical history was taken, and a physical examination was performed. The otolaryngological examination included an assessment of the size of palatine tonsils using the Pirquet scale [19]. The data thus collected determined the allocation to either the tonsillitis (group 1) or tonsillar hypertrophy group (group 2) in the clinical classification. Patients denying episodes of palatine tonsillitis but reporting snoring and/or sleep apnea were in the tonsillar hypertrophy group. The occurrence of episodes of tonsillitis as a single feature or in combination with snoring and/or sleep apnea classified the patient into the tonsillitis group. To extend the study, the environmental factor of tobacco smoking and alcohol consumption was assessed in subjects qualified for surgical treatment. Patients meeting the above criteria were qualified for tonsillectomy.

Inclusion criteria:

1. Palatine tonsillitis diagnosed on the basis of medical history and physical examination, taking into account the SIGN criteria, a minimum of 5 episodes per year, and symptoms for at least one year [20];
2. Conditions of tonsillar hypertrophy based on physical examination and medical history, including OSAS and/or snoring;
3. History of peritonsillar abscess;
4. Adults.

Exclusion criteria:

1. Diagnosed cancer of the palatine tonsils;
2. History of palatine tonsil biopsy;
3. Confirmed or clinically suspected immunodeficiency.

Evaluation of blood parameters

For a more thorough diagnosis of the palatine tonsil diseases in question, blood tests (ASO, ESR, CRP, and levels of leukocytes) were performed in each patient before surgery.

Histopathological assessment

A key step in the pathomorphological diagnosis of palatine tonsil disease was the microscopic evaluation of tonsil specimens to determine morphological markers that differentiate tonsillitis from tonsillar hypertrophy. This examination was performed by a single person to eliminate subjectivity and variations in the evaluation of the examined tissue.

In this study, the condition of tonsillitis and tonsillar hypertrophy diagnosed on the basis of medical history and physical examination (clinical assessment) was compared with the histopathological diagnosis determined by microscopic evaluation of the examined palatine tonsil tissue. This showed which investigated parameters used in the clinical assessment overlapped and which were discrepant with the identical histopathological diagnosis of one of the palatine tonsil conditions.

Since there are no strictly defined histopathological criteria in the available literature to distinguish between chronic tonsillitis and tonsillar hypertrophy, our modification of criteria from the available publications was used [21,22,23,24].

Patients were allocated to one of the pathomorphological groups based on the modified eligibility criteria described in the previous paper [25].

Accordingly, the following were evaluated:

- The number of lymphoid follicles/10 mm^2^: the number of lymphoid follicles > 10/mm^2^;
- Type of lymphoid follicles: divided into (1) primary follicles without germinal centers and (2) secondary follicles with germinal centers;
- Presence of chronic inflammatory infiltration: the presence of individual leukocytes or their groups infiltrating the surface epithelium of the tonsil and subepithelial connective tissue;
- The presence of erosions within the superficial epithelium: Ugras’s abscess—clusters of leukocytes leading to degradation of the epithelium and erosions in its superficial layer;
- The presence of fibrosis;
- The specimens examined were divided into two histopathological groups: (1) tonsilitis and (2) tonsillar hypertrophy.

Immunohistochemical assessment

In the final stage of our study, sections from the resected tonsils were subjected to immunohistochemical assessment using the antibodies IL-1, IL-2, IL-6, IL-8 IL-10, IL-37 and CD25, CD40, and CD69 for each of the study groups, taking into account the division into the following histological regions within the palatine tonsils: epithelial, subepithelial, follicular, follicular center, and interfollicular. The aim was to identify a reliable test that would allow for the differentiation between the two studied groups using the listed immunohistochemical markers.

The slides with the immunohistochemical reactions were scanned using the 3DHISTECH Pannoramic 250 FLASH scanner and evaluated under the Olympus BH63 light microscope. Next, scheduled measurements were made using the 3DHISTECH CaseCenter ver. 2.7 software. In the specimens, the immunohistochemical staining was evaluated in four regions of the tonsil: the lymphoid follicles, the crypt epithelium, the interfollicular region, and the subepithelial region. The phenotypic expression of immunohistochemical reactions was described using a scoring system that considered both staining intensity and the percentage of stained cells. The intensity of the immunohistochemical reaction was assessed on a scale of 0 to 3 (no, weak, moderate, or strong reaction), and the extent was evaluated according to the following criteria < 1% of cells = 0; 1 = 1; >1–10 = 2, 11–33 = 3, 34–66 = 4, 67–100 = 5. The two values were then added together to obtain the result.

Statistical analysis

The data analysis was performed using the GNU PSPP 1.4.1 software. A single-sample test was used to evaluate the significance of determining the mean values. The significance of differences between the parameters of the two groups was established using a *t*-test for independent samples. The significance of differences between the parameters of more than two groups was determined using analysis of variance (ANOVA) with Fisher’s LSD post hoc test. Cochran’s Q test with McNemar’s post hoc analysis was also applied in the statistical analysis.

## 3. Results

**Figure 1:**
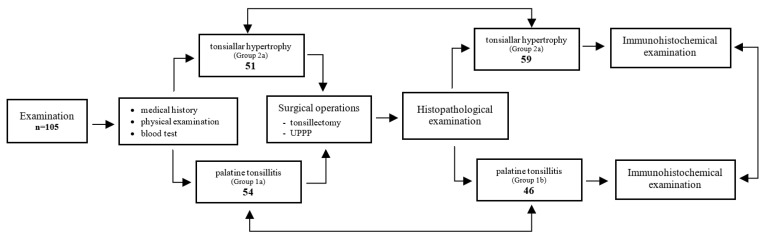
Scheme of the study design.

Due to the independent clinical and pathomorphological assessment of the patients studied, two separate statistical analyses were conducted to evaluate similarities as well as differences in the results obtained. The scheme of the study shown in Figure 1.

The clinical assessment identified 54 patients in the tonsillitis group and 51 in the tonsillar hypertrophy group. However, in the pathomorphological assessment, group 1b contained 46, while group 2b contained 59 patients. The results are shown in Table 1.

When evaluating the clinical assessment (assuming the histopathological diagnosis is correct), the following is necessary:

Specificity: 47%, sensitivity: 50%.

When evaluating the histopathological assessment (assuming the clinical diagnosis is correct), the following in necessary:

Specificity: 55%, sensitivity: 43%.

Clinical assessment: physical examination

The clinical assessment yielded statistically significant results (*p* < 0.05) for the size of the palatine tonsils. Larger tonsils were found in the tonsillitis group. The mean palatine tonsil size was 2.78 for the tonsillar hypertrophy group and 3.17 for the tonsillitis group. According to the Pirquet scale, the size of the palatine tonsils in both study groups ranged from grade 1 to grade 5. The results are shown in Table 2.

There were 54 patients in the palatine tonsillitis group and 51 patients in the palatine tonsillar hypertrophy group. A higher mean age of 44.7 years occurred for the palatine tonsillar hypertrophy group compared to the palatine tonsillitis group. The patients were aged from 19 to 70 years. The majority of patients in the palatine tonsillar hypertrophy group were male—45 patients (88%), while in the palatine tonsillitis group, men accounted for 50% (27 patients). In the clinical assessment, 11 patients of the palatine tonsillitis group (20.4%) and 13 patients of the palatine hypertrophy group (25.5%) smoked tobacco products. No statistical significance was found at *p* < 0.05. Similar results were obtained among subjects reporting alcohol consumption. In the palatine tonsillitis group, this was reported in 15 patients and in the palatine tonsillar hypertrophy group in 17 patients. The analysis also showed no statistically significant differences.

Clinical assessment: Blood test

To deepen the diagnosis of tonsillar diseases, inflammatory markers (ASO, ESR, CRP, and leukocytes) were compared within the studied groups. In the clinical assessment, statistically significant differences were obtained only for ASO at *p* < 0.05. Its level was higher in the tonsillitis group and amounted to 194.85 compared to the tonsillar hypertrophy group, where a level of 122.07 was recorded. The mean value of ESR was slightly higher in the tonsillar hypertrophy group, with a result of 4.75. In the tonsillitis group, its level was 4.72. CRP was slightly higher in the tonsillitis group with a score of 0.38 compared to the tonsillar hypertrophy group, where it was 0.22. Leukocytosis was more elevated in the tonsillar hypertrophy group than in the tonsillitis group, the results being 7.26 and 6.70, respectively. The results are shown in Table 3.

Pathomorphological assessment: Physical examination

In the pathomorphological assessment, statistically significant results (*p* < 0.05) were obtained for the size of the tonsils between the two examined groups. Larger tonsils were found in the tonsillar hypertrophy group. Their mean size was 3.19 for the tonsillar hypertrophy group and 2.72 for the tonsillitis group. According to the Pirquet scale, the size of the palatine tonsils in the tonsillitis group ranged from grade 1 to grade 4, while in the tonsillar hypertrophy group, the smallest tonsils according to the Pirquet scale were grade 2, and the largest were grade 5. The results are shown in Table 4.

There were 46 patients in the palatine tonsillar hypertrophy group and 59 in the palatine tonsillitis group. The mean age was higher for the palatine tonsillitis group than for the palatine tonsillar hypertrophy group; it was 45.17 years for the palatine tonsillar hypertrophy group and 35.44 years for the palatine tonsillar hypertrophy group. The patients’ age ranged from 19 to 70 years. The majority of subjects, irrespective of allocation, were male. They represented 72% of the palatine tonsillitis group (33 patients) and 66% of the palatine hypertrophy group (39 patients). Women accounted for 28%, i.e., 13 patients, of the palatine tonsillitis group and 34%, i.e., 20 patients, of the palatine tonsillar hypertrophy group. Seven patients of the palatine tonsillitis group (15.2%) and seventeen (28.8%) of the palatine tonsillar hypertrophy group smoked tobacco products, while alcohol consumption was reported in twelve patients of the palatine tonsillitis group and in fifteen patients of the palatine tonsillar hypertrophy group. For both smoking and alcohol consumption, there were no statistically significant differences at *p* < 0.05.

Pathomorphological assessment: Blood test

To analyze tonsil diseases, the values of ASO, ESR, CRP, and leukocyte levels were compared within the study groups. The ASO level in the tonsillar hypertrophy group was 163.8 and was higher than in the tonsillitis group, where a value of 157.57 was recorded. The mean value of ESR was slightly higher in the tonsillitis group with a result of 5.00, while in the tonsillar hypertrophy group, a result of 4.51 was recorded. CRP was higher in the tonsillar hypertrophy group with a result of 0.39 compared to the tonsillitis group, where it was 0.2. The leukocyte levels were almost identical in both study groups. In the tonsillitis group, it was 6.93 and in the tonsillar hypertrophy group 6.98. No statistically significant correlation was found for the indicators of inflammation. Detailed results are presented in Table 5.

The isolated values of inflammatory parameters have limited diagnostic value. Our analysis aimed to determine a profile of ASO, ESR, CRP, and leukocyte levels that could be characteristic diagnostic parameters of one of the two tonsil diseases.

In the pathomorphological assessment, 75% of the examined individuals who had elevated ESR (>4.73) and leukocytosis (>6.96) as well as decreased ASO (<161.03) and CRP (<0.31) belonged to the tonsillitis group. The results are presented in Table 6.

Pathomorphological assessment: Histopathological assessment

Based on the pathomorphological assessment, statistically significant differences were noted for the following parameters that were used to classify diseases of the palatine tonsils: number of follicles, number of germinal centers, fibrosis, the thickness of subepithelial fibrosis, number of follicles per crypt, and crypt thickness. The average number of follicles was higher in the tonsillar hypertrophy group with a result of 13.98 compared to the tonsillitis group, whose value was 9.13. The average number of centers was also higher in the tonsillar hypertrophy group than in the tonsillitis group. It was 9.97 for the tonsillar hypertrophy group and 5.76 for the tonsillitis group. The level of fibrosis was significantly higher in the tonsillitis group, reaching a result of 65.2% compared to the tonsillar hypertrophy group with a result of 22%. The mean thickness of the subepithelial fibrosis layer was higher in the tonsillitis group, reaching 376.3, while in the tonsillar hypertrophy group, it was 248.46. The average level of inflammatory infiltration was higher in the tonsillitis group, reaching 82.6%, while in the tonsillar hypertrophy group, it was 69.5%. The number of Ugras’s abscesses was slightly higher in the tonsillar hypertrophy group with a result of 25 (42.4%) compared to the tonsillitis group, where a total of 19 (41.3%) was observed. The number of actinomyces was similar in both groups. In the tonsillar hypertrophy group, it was 39.0% and in the tonsillitis group 43.5%. The number of follicles on the surface of the crypt was higher in the tonsillar hypertrophy group, amounting to 17.29 compared to the tonsillitis group, where it was 10.76. The average thickness of the crypt was also higher in the tonsillar hypertrophy group with a value of 1222.73 compared to the tonsillitis group, where its measured value was 1038.33. The average thickness of the crypt epithelium in the tonsillar hypertrophy group was 178.73 and was lower than in the tonsillitis group, where it was 206.17. The results are shown in Table 7 and Table 8.

Comparison of clinical and pathomorphological assessment

It has been shown that the measured values of ASO, ESR, CRP, and leukocytosis level did not differ significantly between the tonsillitis and tonsillar hypertrophy groups in the pathomorphological assessment regardless of the adopted clinical classification. The investigated parameters did not provide a useful criterion that correlated with the pathomorphological and clinical assessments. Statistically significant differences between the tonsillitis and the tonsillar hypertrophy groups were observed for the tonsil size. A higher value of 3.07 was obtained in the tonsillar hypertrophy group compared to the tonsillitis group, for which the value was 2.43. Detailed results are presented in Table 9.

Immunohistochemical assessment

The region of the tonsil tissue where the most statistically significant differences were observed between the studied markers was the follicular center, where CD-40 and IL-1 responses were observed. The highest statistically significant value was shown by IL-1, which was 1.95 in the tonsillar hypertrophy group. The lowest statistically significant value was 0.42, observed for CD-40 in the tonsillar hypertrophy group. The epithelial, follicular, and interfollicular regions showed only one statistically significant value for the studied chemokines and lymphokines. For the epithelial region, IL-10 demonstrated a response of 0.26 for the tonsillitis group and 0.29 for the tonsillar hypertrophy group. CD-25 showed a response for the follicular and interfollicular regions, reaching a value of 0.8 for the follicular region in the tonsillitis group and 0.035 in the tonsillar hypertrophy group. In the interfollicular region, the CD-25 value was that of 1.22 in the tonsillitis group and 0.51 in the tonsillar hypertrophy group. However, the subepithelial region was the only region where no statistically significant differences with *p* < 0.05 between the studied groups were noted. Characteristically, only in this region WAS the highest level of all tested markers observed. It was that of IL-8, with a result of 5.08 in the tonsillar hypertrophy group. It should be emphasized that for the data encompassing the entire sample (calculated on the basis of measurements for all regions combined), no statistically significant differences were noted. In both groups, the highest concentration was observed for IL-8 and the lowest for IL-10. Thus, the obtained relationships are convergent within the analyzed locations. The results, including detailed calculation values, are presented in Table 10.

## 4. Discussion

In the study conducted by Mikola et al. [26], expression of IL-10 and IL-37 was detected in the tissue of the palatine tonsil. In the tissue affected by inflammation, the average value of IL-10 expression was lower than in the tissue of the hypertrophic palatine tonsil. Our study obtained consistent results. It should be emphasized, however, that in contrast to our study, the authors did not identify the region of the tonsillar tissue with the highest expression of this cytokine. In addition, the differences found were not statistically significant. In our study, we introduced this division and found the highest expression of this chemokine in the subepithelial region for the two studied groups: 0.54 for the tonsillar hypertrophy group and 0.37 for the tonsillitis group. It should be noted that the only region with statistically significant expression levels of this chemokine was the epithelial region. As the authors themselves emphasize, among the cytokines studied, only the newly discovered anti-inflammatory interleukin IL-37 was independently associated with the hypertrophy of the palatine tonsils, showing a slightly stronger anti-inflammatory response in these patients. Our analysis also revealed a higher level of IL-37 expression in the tissue affected by hypertrophy. The authors of the cited publication suggest that hypertrophy of the palatine tonsils may be a consequence of chronic tonsillitis, which would indicate a blurring of the boundaries between the levels of cytokine expression, including IL-10, in both diseases.

Huang et al. [27] found expression of IL-1, IL-6, and IL-10 in tissue affected by tonsillar hypertrophy as well as tonsillar hypertrophy with concurrent tonsillitis. The researchers demonstrated a higher level of expression of the examined cytokines in the isolated hypertrophy group. In our study, a higher expression of IL-6 was observed in the group with tonsillitis. However, the levels of expression of IL-1 and IL-10 were higher in the tonsillar hypertrophy group, consistent with the results described in the cited publication. As the authors of the publication emphasize, the etiology of the hypertrophy of the lymphoid tissue of the palatine tonsils remains unknown to this day. The cited study detected high VP1 expression in hypertrophic tonsils, indicating that it may be caused by viral infection. Partially divergent results obtained by the authors of the aforementioned publication compared to ours may be due to the lack of a uniformly isolated group with palatine tonsillitis. Comparing cytokine expression profiles of two groups, of which one has the same component as the first, appears to be imprecise and carries a high risk of misjudgment. Our study identified two separate groups, which allowed for obtaining independent results with statistically significant values for selected components.

Geißler et al. [28] investigated the ability of T lymphocytes in tonsillitis to maintain a high basal level of expression of surface markers CD25, CD69, and CD154 (CD40) in freshly isolated T lymphocytes from the tonsils. The results obtained in patients with tonsillitis were characterized by higher expression of CD25, CD69, and CD154 (CD40) compared to peritonsillar abscess and tonsil hypertrophy. Our study demonstrated consistent results for CD25 and CD40. However, the expression level of CD69 was higher in the group with tonsillar hypertrophy. The levels of CD25, CD40, and CD69 for all tissues examined were as follows: for the group with tonsillitis, 1.40, 0.60, and 1.34, and for the group with tonsillar hypertrophy, 1.04, 0.5, and 1.46, respectively. Our study also identified regions of the palatine tonsil with high expression of the above-mentioned markers. The highest values were obtained in the subepithelial region for CD69. CD40 showed statistically significant differences in the follicular center region of the palatine tonsils. However, CD25 demonstrated statistically significant correlations in the follicular and interfollicular regions between the analyzed groups. As emphasized by the authors of the above-mentioned publication, T cells in tonsillitis show signs of increased basal activation, which is reflected in high basal expression of surface activation markers such as CD69, CD40, and CD25. This is consistent with the view that tonsillar T cells are exposed to chronic stimulation. However, chronic antigen exposure, initially conducive to the accumulation of pathogen-specific effector T cells, may ultimately lead to the exhaustion of the effector T-cell population [29,30,31,32].

Guo Chen et al. [33] compared two groups of children: those with OSAS and without tonsillitis (OSAS group) with a control group consisting of children with tonsillitis without accompanying OSAS. They found higher expression of IL-1 in the OSAS group compared to the control group. In addition, the expression of IL-1 in the tonsillar tissue showed higher concentrations in the medullary than in the cortical zone. In our analysis, the level of IL-1 in the tonsillar hypertrophy group in specific regions was as follows: epithelial, 1.32; subepithelial, 4.32; follicular, 0; follicular center, 1.95; and interfollicular, 2.76. Its overall level in our study was also higher in the tonsillar hypertrophy group. The subepithelial and follicular center regions showed higher expression levels in the tonsillar hypertrophy group than in the tonsillitis group. However, the follicular center was the only region that showed statistically significant differences in favor of hypertrophic tonsils. The authors of the publication also compared the concentration of IL-10 between the groups. The OSAS group showed a higher level of IL-10 than the control group. Our analysis revealed the same relationship. The higher level of IL-6 in the group with tonsillitis reported by the authors of the publication is consistent with our findings. However, the authors’ finding of a higher expression of IL-8 in the tonsil tissue affected by hypertrophy is opposite to our results. The cited study [33] and our study included the palatine tonsils but did not account for the adenoids, which also play a role in the pathophysiology of OSAS and are associated with inflammatory markers related to sleep disorders. In addition, the authors only studied a pediatric population aged 3 to 12 years and included only thirty-four patients. In our study, the group of patients is more diverse in terms of age range and the number of cases analyzed. This may be a basis for the differences in results for the cytokines studied between the two groups.

The next particularly important publication [34] compared the values of antistreptolysin O in different tonsillar diseases in adult patients. In the group with chronic tonsillitis, the mean ASO titer was 363, which was significantly higher compared to acute tonsillitis. In our analysis, we also determined its level to demonstrate whether the ASO titer can serve as an indicator for differentiating tonsillar diseases. In the available literature, it has been accepted that levels above 333 IU/mL should be considered abnormal [35]. However, in a more recent study, it was shown that the reference serum values for ASO show a large deviation from the currently used reference value (200 IU/mL). The authors suggest that reference values for ASO should be established for specific ethnic groups and regions [36]. The laboratory that performed the measurements in our study assumed the standardized reference value of 200 IU/mL to be the upper limit of normal. The first stage of the study confirmed the significance of ASO values for cases of acute tonsillitis in adults. Our analysis showed differences in ASO levels between the studied groups of tonsillar diseases. In the clinical assessment, statistically significant differences were also found between the tonsillitis and tonsillar hypertrophy groups in favor of the group with tonsillitis. However, the obtained ASO values are within the range of the current norm, both for clinical and pathomorphological assessment. Therefore, it should be stated that this parameter may be an ancillary factor in the diagnosis of tonsillar diseases. The adopted standardized value of the ASO level does not allow for the identification of an abnormal state in our research. The data regarding the level of ASO antibodies in our study have a higher value in clinical than in pathomorphological assessment, as a statistically significant difference was obtained. In summary, our analysis confirms the concept of the significant role of the ASO parameter in tonsillar diseases. The limitation of our investigation and that of the aforementioned study is its confidence level conditioned by the standardized value of ASO. The normative data for antistreptolysin O are specific to individual populations due to differences in the epidemiology of GABHS between populations. Therefore, conclusions regarding the highest values may only be applicable to the general population after calibrating them to local conditions.

CRP is a parameter that increases rapidly in inflammatory conditions. This protein can also be found in trace amounts in the blood of patients without symptoms of inflammation [37]. Given that a positive correlation has been found between the size of the tonsils and the number of B and T cells [38], we decided to analyze this parameter more extensively in our study. Unfortunately, as in the case of ASO, no statistically significant differences between the study groups were observed. This suggests a low diagnostic value of CRP as an isolated marker in detecting tonsil diseases.

The white blood cell count (WBC) and ESR are other parameters characteristic of inflammatory diseases. Lopez-Gonzales et al. [39] regarded tonsillar hypertrophy without accompanying inflammation as a disease with a distinct basis of changes occurring in the tonsil tissue, potentially differing in the intensity of immune reactions taking place within the tissue. Variations in the number of leukocytes in the blood were expected to be statistically significant in the groups studied. Characteristically, the results were consistent both in the publication cited and in our findings. This reinforces our conviction of the lack of predictive value of a single marker in the diagnosis of diseases of the palatine tonsils.

In the study by Reichel et al. [40], the authors speculated that ASO levels would indicate significantly higher values in palatine tonsillitis compared to palatine tonsillar hypertrophy. As in our study, there was no statistically significant difference above the normative values in serum anti-ASO antibody levels between the palatine tonsil diseases studied. Yokoyama et al. [41] also found no differences in ASO levels between the population of children with palatine tonsillitis and those without an inflammatory process. In conclusion, ASO values should be analyzed together with other parameters of inflammation because as an isolated factor alone, it does not show a high positive predictive value.

Our study showed that preoperative laboratory tests for isolated inflammatory markers did not show significant differences between patients with palatine tonsillar hypertrophy and palatine tonsillitis. Only the combination of the four inflammatory parameters—ASO, ESR, CRP, and leukocytosis level—developed by us and discussed in more detail in the conclusions showed a predictive value for differentiating the palatine tonsil diseases studied.

The pathomorphological examination conducted by Ahmed et al. [42] aimed to determine the morphological features characterizing the clinical state of inflammation in the tonsils on histological sections. Lymphoid follicles with proliferation, lymphoid hyperplasia, spongiosis of the tonsillar tissue, and lymphocytic infiltration were observed. Our study revealed an increase in lymphocytic infiltration and an elevated number of follicles in the tonsillar hypertrophy group compared to the tonsillitis group. In addition, our study research expanded the analysis to include the histological categories of the palatine tonsil tissue (epithelial, subepithelial, follicular, follicular center, and interfollicular). The observed similarities and differences are presented in detail in the conclusions. It should be emphasized that the aforementioned study had the following major limitations: the homonymous group of disorders of palatine tonsil homeostasis, a small group included in the study, and the fact that only the pediatric population was analyzed. Our research took into account all of these shortcomings, which makes the analysis more diagnostically valuable. In summary, a comprehensive evaluation of the microscopic image of palatine tonsil tissue indicates a multifaceted combination of cellular processes occurring within it, resulting in a given histological picture.

The growing efforts in recent years to understand the immunological functions occurring in the palatine tonsils have led to a debate on the validity of tonsillectomy and tonsillotomy [43,44]. Our novel tonsillectomy system illustrates the complexity of immune processes in the tissue of the palatine tonsil as well as the complexity of its structure. At the same time, our detailed analysis makes it possible to identify with a high degree of certainty the type of tissue abnormality of the palatine tonsil that is an indication for tonsillectomy/tonsillotomy. Our data underline the view that the palatine tonsils are functional immune units and suggest that tonsillotomy, i.e., partial resection of the palatine tonsils, should be considered in the future as an alternative to total tonsillectomy (tonsillectomy) in adults [45,46].

## 5. Conclusions

1. The present study shows significant discrepancies in the clinical and pathomorphological assessment of tonsils undergoing tonsillectomy, which is why these methods should be considered complementary to each other;
2. Medical history and physical examination, depending on the adopted clinical or histopathological classification, show a differentiation in the distribution of features that form the basis of allocation to a particular group;
3. The diagnostic value of blood tests including ASO, ESR, CRP, and leukocytosis proved to be a significant predictive factor for tonsil diseases;
4. The conducted pathomorphological assessment revealed a diverse profile of expression of the investigated immunohistochemical markers depending on the diagnosed tonsil disease.

## Figures and Tables

**Table 1:** Comparison of the distribution of patients of the tonsillitis and tonsillar hypertrophy groups according to the classification criterion adopted, i.e., clinical or pathomorphological assessment.

		Histopathological Assessment	
		Tonsillitis	Tonsillar Hypertrophy
Clinical assessment	Tonsillitis	23 (43%) *	31 (57%) *
	Tonsillar hypertrophy	23 (45%) *	28 (55%) *

Values shown in the table do not differ at the significance level *p* < 0.05 between the tonsilitis and tonsillar hypertrophy groups; the score with “*” % of patients in pathomorphological assessment.

**Table 2:** The size of palatine tonsils for the tonsillitis and tonsillar hypertrophy groups in clinical assessment.

Study Group	Size of Tonsils				
	Mean	*p*-Value	Min.	Max.	SD
Tonsillitis Group 1a	**3.17**	<0.05	1	5	0.833
Tonsillar hypertrophy Group 2a	**2.78**	<0.05	1	5	0.832

Bold text: values that differ significantly at the level of *p* < 0.05 between the tonsillitis and tonsillar hypertrophy groups. M, mean; SD, standard deviation; Min. and Max., minimum and maximum values of the distribution.

**Table 3:** Values of ASO, ESR, CRP, and leukocytosis for the tonsillitis group and the tonsillar hypertrophy group in clinical assessment.

Study Group	M	Mdn.	SD	Min.	Max.	Q1	Q3	*p*-Value
Tonsillitis (Group 1a)								
ASO	**194.85**	106.00	210.19	0	840	54	262	<0.05
ESR	4.72	4.00	3.19	1	14	2	6	<0.05
CRP	0.38	0.10	1.26	0	9	0	0	<0.05
Leukocytosis	6.70	6.59	1.78	3.7	12.2	5.4	7.8	<0.05
Tonsillar hypertrophy (Group 2a)								
ASO	**122.07**	95.50	118.00	0	544	31	171	<0.05
ESR	4.75	4.00	3.56	1	17	2	6	<0.05
CRP	0.22	0.10	0.35	0	2	0	0	<0.05
Leukocytosis	7.26	6.73	2.02	4.8	13.2	5.8	7.8	<0.05

Bold text: values that differ significantly at the level of *p* < 0.05 between the tonsillitis and tonsillar hypertrophy groups. M, mean; Mdn., median; SD, standard deviation; Min. and Max., minimum and maximum values of the distribution; Q1 and Q3, first and third quartiles.

**Table 4:** The size of palatine tonsils for the tonsillitis group and the tonsillar hypertrophy group in pathomorphological evaluation.

Study Group	Size of Tonsils			
	M	*p*-Value	Min.	Max.
Tonsillitis Group 1b	**2.72**	<0.05	1	4
Tonsillar hypertrophy Group 2b	**3.19**	<0.05	2	5

Bold text: values that differ significantly at the level of *p* < 0.05 between the tonsillitis and tonsillar hypertrophy groups. M, mean; Mdn., median; SD, standard deviation; Min. and Max., minimum and maximum values of the distribution.

**Table 5:** Values of ASO, ESR, CRP, and leukocytosis for the tonsillitis group and the tonsillar hypertrophy group in pathomorphological assessment.

Study Group	M	Mdn.	SD	Min.	Max.	Q1	Q3	*p*-Value
Tonsillitis (Group 1b)								
ASO	157.57	105.50	183.86	0	840	52	178	<0.05
ESR	5.00	5.00	3.05	1	14	3	7	<0.05
CRP	0.20	0.10	0.39	0	2	0	0	<0.05
Leukocytosis	6.93	6.62	1.86	4.3	12.7	5.6	7.8	<0.05
Tonsillar hypertrophy (Group 2b)								
ASO	163.80	89.00	172.13	0	774	40	239	<0.05
ESR	4.51	4.00	3.59	1	17	2	5	<0.05
CRP	0.39	0.20	1.23	0	9	0	0	<0.05
Leukocytosis	6.98	6.71	1.96	3.7	13.2	5.7	7.8	<0.05

M, mean; Mdn., median; SD, standard deviation; Min. and Max., minimum and maximum values of the distribution; Q1 and Q3, first and third quartiles.

**Table 6:** Percentage distribution of results for parameters meeting the following conditions: ESR (>4.73), leukocytosis (>6.96), ASO (<161.03), and CRP (<0.31) in the pathomorphological assessment.

Study Group		Simultaneous ESR > 4.73, Leukocytosis > 6.96, ASO < 161.03, and CRP < 0.31		Total	Cramer’s V
		Positive	Negative		
Tonsillitis Group 1b	Counts	9	35	44	0.23
	% in rows	20.5%	79.5%	100%	
	% in columns	75.0%	40.2%	44.4%	
	% of total	9.1%	35.4%	44.4%	
Tonsillar hypertrophy Group 2b	Counts	3	52	55	
	% in rows	5.5%	94.5%	100%	
	% in columns	25.0%	59.8%	55.6%	
	% of total	3.0%	52.5%	55.6%	
Total	Counts	12	87	99	
	% in rows	12.1%	87.9%	100%	
	% in columns	100%	100%	100%	
	% of total	12.1%	87.9%	100%	

Demonstrated for ESR test (>4.73), leukocytosis (>6.96), ASO (<161.03), and CRP (<0.31): specificity 79.5%, sensitivity: 5.5%.

**Table 7:** Histological features of palatine tonsils in pathomorphological assessment in the tonsillitis group and the tonsillar hypertrophy group.

Study Group	Number of Follicles		Number of Germinal Centers		Fibrosis		Thickness of Subepithelial Fibrosis Layer		Inflammatory Infiltration		Ugras’s Abscesses	
	Mean	*p*-Value	Mean	*p*-Value	Quantity	V *	Mean	*p*-Value	Quantity	V *	Quantity	V *
Tonsillitis Group 1b	**9.13**	<0.05	**5.76**	<0.05	**30 (65.2%)**	0.44	**376.30**	<0.05	38 (82.6%)	-	19 (41.3%)	-
Tonsillar hypertrophy Group 2b	**13.98**	<0.05	**9.97**	<0.05	**13 (22.0%)**		**248.46**	<0.05	41 (69.5%)		25 (42.4%)	

* Cramer’s V coefficient. Bold text: values that differ significantly at the level of *p* < 0.05 between the tonsillitis and tonsillar hypertrophy groups.

**Table 8:** Histological features of palatine tonsils in pathomorphological assessment in the tonsillitis group and the tonsillar hypertrophy group.

Study Group	Number of Actinomyces		Number of Follicles per Crypt		Thickness of the Crypt		Thickness of the Crypt Epithelium	
	Quantity	V *	Mean	*p*-Value	Mean	*p*-Value	Mean	*p*-Value
Tonsillitis Group 1b	20 (43.5%)	-	**10.76**	<0.05	**1038.33**	<0.05	206.17	<0.05
Tonsillar hypertrophy Group 2b	23 (39.0%)		**17.29**	<0.05	**1222.73**	<0.05	178.73	<0.05

* Cramer’s V coefficient. Bold text: values that differ significantly at the level of *p* < 0.05 between the tonsillitis and tonsillar hypertrophy groups.

**Table 9:** Comparison of ASO, ESR, CRP, leukocytosis, and tonsil size between the tonsillitis and the tonsillar hypertrophy groups in clinical and pathomorphological assessment, showing the degree of convergence of diagnoses relative to a given group.

Study Group	Histopathological Group	ASO		ESR		CRP		Leukocytosis		Size of Tonsils			
		Mean	*p*-Value	Mean	*p*-Value	Mean	*p*-Value	Mean	*p*-Value	Mean	*p*-Value	Min.	Max.
tonsillitis	Tonsillitis	194.83	<0.05	4.74	<0.05	0.19	<0.05	6.49	<0.05	3.00	<0.05	1	4
	Tonsillar hypertrophy	194.87	<0.05	4.70	<0.05	0.53	<0.05	6.86	<0.05	3.29	<0.05	2	5
tonsillar hypertrophy	Tonsillitis	116.76	<0.05	5.29	<0.05	0.21	<0.05	7.41	<0.05	**2.43**	<0.05	1	4
	Tonsillar hypertrophy	126.52	<0.05	4.28	<0.05	0.22	<0.05	7.13	<0.05	**3.07**	<0.05	2	5

Bold text: values that differ significantly at the level of *p* < 0.05 between the tonsillitis and tonsillar hypertrophy groups.

**Table 10:** Results of immunohistochemical reaction measurements of the tonsil in the tonsillitis and tonsillar hypertrophy groups, considering individual tissue regions and the entire palatine tonsil in pathological evaluation.

		Epithelial		Subepithelial		Follicular		Follicular Center		Interfollicular		Total *	
		Mean	*p*-Value	Mean	*p*-Value	Mean	*p*-Value	Mean	*p*-Value	Mean	*p*-Value	Mean	*p*-Value
Tonsillitis Group 1b	CD-25	1.11	<0.05	2.43	<0.05	**0.8**	<0.05	1.41	<0.05	**1.22**	<0.05	1.4	<0.05
	CD-40	0.33	<0.05	0.91	<0.05	0.045	<0.05	**1.35**	<0.05	0.37	<0.05	0.6	<0.05
	CD-69	1.07	<0.05	3.17	<0.05	0	-	1.65	<0.05	0.83	<0.05	1.34	<0.05
	IL-1	1.41	<0.05	4.22	<0.05	0.09	<0.05	**0.98**	<0.05	2.93	<0.05	1.93	<0.05
	IL-10	**0.26**	<0.05	0.37	<0.05	0	-	0.22	<0.05	0.2	<0.05	0.21	<0.05
	IL-2	0.43	<0.05	1.11	<0.05	0	-	0.22	<0.05	0.5	<0.05	0.45	<0.05
	IL-37	0.30	<0.05	2.48	<0.05	0.24	<0.05	0.74	<0.05	1.28	<0.05	1.01	<0.05
	IL-6	1.78	<0.05	2.52	<0.05	0.17	<0.05	1.15	<0.05	0.54	<0.05	1.23	<0.05
	IL-8	0.54	<0.05	5.07	<0.05	4.76	<0.05	2.96	<0.05	4.5	<0.05	3.57	<0.05
Tonsillar hypertrophy Group 2b	CD-25	1.03	<0.05	2.46	<0.05	**0.035**	<0.05	1.15	<0.05	**0.51**	<0.05	1.04	<0.05
	CD-40	0.51	<0.05	1.44	<0.05	0	-	**0.42**	<0.05	0.14	<0.05	0.5	<0.05
	CD-69	1.22	<0.05	3.37	<0.05	0	-	1.66	<0.05	1.07	<0.05	1.46	<0.05
	IL-1	1.32	<0.05	4.32	<0.05	0	-	**1.95**	<0.05	2.76	<0.05	2.07	<0.05
	IL-10	**0.29**	<0.05	0.54	<0.05	0	-	0.19	<0.05	0.17	<0.05	0.24	<0.05
	IL-2	0.53	<0.05	1.34	<0.05	0	-	0.15	<0.05	0.56	<0.05	0.515	<0.05
	IL-37	0.59	<0.05	3.17	<0.05	0	-	1.2	<0.05	0.92	<0.05	1.18	<0.05
	IL-6	2.03	<0.05	2.49	<0.05	0.035	<0.05	0.9	<0.05	0.32	<0.05	1.16	<0.05
	IL-8	0.31	<0.05	5.08	<0.05	4.32	<0.05	2.47	<0.05	4.02	<0.05	3.24	<0.05

* The overall value is calculated as the average of all measurements. Bolded values differ statistically at *p* < 0.05 between the tonsillitis and tonsillar hypertrophy groups.

## Data Availability

The data analyzed in study are present in Department of Otolaryngology and Cranio-Maxillo-Facial Surgery and Department of Pathomorphology, Military Institute of Medicine—National Research Institute.
